# How effective are family-based and institutional nutrition interventions in improving children’s diet and health? A systematic review

**DOI:** 10.1186/s12889-017-4795-5

**Published:** 2017-10-17

**Authors:** Andrew P. Black, Katina D’Onise, Robyn McDermott, Hassan Vally, Kerin O’Dea

**Affiliations:** 1 School of Health Sciences, University of South Australia, Adelaide, South Australia Australia; 2 Bulgarr Ngaru Medical Aboriginal Corporation, PO Box 170, South Grafton, NSW 2460 Australia; 3 College of Public Health, Medical and Veterinary Sciences, James Cook University, Cairns, QLD Australia; 4 School of Psychology & Public Health, La Trobe University, Melbourne, VIC Australia

**Keywords:** Children, Nutrition programs, Family-based, School/Preschool

## Abstract

**Background:**

Effective strategies to improve dietary intake in young children are a priority to reduce the high prevalence of chronic non-communicable diseases in adulthood. This study aimed to assess the impact of family-based and school/preschool nutrition programs on the health of children aged 12 or younger, including the sustainability of these impacts and the relevance to socio-economic inequalities.

**Methods:**

A systematic review of literature published from 1980 to December 2014 was undertaken. Randomised controlled trials involving families with children aged up to 12 years in high income countries were included. The primary outcomes were dietary intake and health status. Results were presented in a narrative synthesis due to the heterogeneity of the interventions and outcomes.

**Results:**

The systematic search and assessment identified 39 eligible studies. 82% of these studies were set in school/preschools. Only one school study assessed the impact of involving parents systematically. The family-based programs which provided simple positive dietary advice to parents and regular follow-up reduced fat intake significantly. School and family-based studies, if designed and implemented well, increased F&V intake, particularly fruit. Effective school-based programs have incorporated role-models including peers, teachers and heroic figures, rewards and increased access to healthy foods. School nutrition programs in disadvantaged communities were as effective as programs in other communities.

**Conclusions:**

Family and school nutrition programs can improve dietary intake, however evidence of the long-term sustainability of these impacts is limited. The modest overall impact of even these successful programs suggest complementary nutrition interventions are needed to build a supportive environment for healthy eating generally.

**Electronic supplementary material:**

The online version of this article (10.1186/s12889-017-4795-5) contains supplementary material, which is available to authorized users.

## Background

Despite increased interest in food and cooking, high levels of sub-optimal dietary behaviour have been documented in many countries. Nutrition surveys report low levels of fruit and vegetable intake, inadequate intake of important nutrients and high intake of energy-dense nutrient poor (EDNP) foods in all age-groups [1–8]. Less healthy dietary behaviour is an important factor underlying the high prevalence of chronic non-communicable diseases, including cardiovascular disease and diabetes mellitus, and increasing obesity rates [9–11]. Early signs of these chronic diseases and risk factors are increasingly being identified in children and adolescents [12, 13]. This emphasises the importance of promoting and supporting the development of healthy eating habits from an early age.

Thus, a key challenge is how to support and engage families to make sustainable changes to healthier eating, despite the challenges posed within contemporary society. Preschool and childcare are increasingly central in family life, in addition to the importance of schools. Given the ready access to children, these settings have been widely used in programs which aim to improve the dietary intake of young children [14]. These nutrition programs have included the reintroduction and expansion of school meal programs, healthy lifestyle promotion programs, economic incentives, enhancement of school canteens and restrictions on the availability of less healthy foods within schools. Previous reviews found that school-based nutrition programs are moderately effective at improving diet quality, reducing adiposity and improving fruit intake in the short-term [15–17]. Importantly, programs in preschools and schools also offer opportunities to address social inequality by improving and/or targeting the involvement of disadvantaged students.

In addition, many of these institutional programs have also incorporated strategies to engage with families to strengthen their impact, as this is seen as vital to improving the sustainability of healthier eating patterns in the community [15, 18]. The emphasis on the role of the family in promoting healthy eating is underpinned by an understanding of the social and cultural context which shapes the preparation, sharing and eating of food [19]. Healthy changes to an individual child’s eating patterns will be reinforced if the family also shares the same influences and models the changes in behaviour [20]. The question that this poses is how to influence the dietary patterns in the family. Notwithstanding the major role that women have traditionally had in food preparation, fathers and children have also been shown to have an important impact on family food choices [21]. Current research has been increasingly focussed on reducing the prevalence of childhood obesity and thus nutrition interventions have frequently been part of multi-component healthy lifestyle programs [15]. Golley et al. have identified features associated with more effective engagement of parents in child obesity prevention programs including greater parent involvement in implementation, use of self-monitoring and goal-setting behaviour change techniques [22, 23]. More recent reviews of child obesity prevention interventions have highlighted that programs of longer duration involving both physical activity and diet strategies in both schools and home/community settings are more likely to produce sustainable improvements in body mass index (BMI) [24, 25]. Most of these child obesity prevention studies involved children <12 years [15, 24].

However, the evidence of the optimal strategies to involve parents in the promotion of healthier eating in the family is limited. In a 2012 systematic review of school nutrition programs, van Lippenwelde et al. found insufficient evidence from RCTs to explicitly assess the effectiveness of parental involvement [26]. A 2010 systematic review of both school and community child nutrition programs, also concluded that well designed studies are still needed to assess the effectiveness of parental involvement in these programs [27]. Family based interventions have been shown to be more effective in children <12 years in a systematic review of childhood obesity treatment [28]. Thus, the objective of this systematic review of randomised controlled trials was to document the potential for family-based interventions to complement institutional nutrition programs to improve the nutrition and health of young children in high income countries. Another aim was to determine how sustainable the impacts of these nutrition programs are on children’s nutrition and health. Finally, lower socio-economic status is associated with both higher risk of chronic non-communicable disease and lower uptake of health promoting behaviours, including healthy eating. Thus, the impact of these nutrition programs that may help to reduce this social inequality was also reviewed.

## Methods

The review was undertaken using the principles outlined in the Cochrane Handbook [29] and the Cochrane Public Health and Health Promotion guidelines [30] with the exception that only one reviewer scanned the title list and/or abstract and that only studies published in English were included. The following definitions were used to identify relevant studies:

Family-Based programs- all programs which involved one or more adults with or without their children in any setting.

Institutional programs- programs involving groups of children in organised settings such as schools, pre-schools, childcare including out of school hours care, youth and church groups.

Nutrition program- all interventions where one major aim was to improve the quality of dietary intake.

Criteria for inclusion in the review:

Types of study

Randomised controlled trials (RCTs) including cluster randomised trials were eligible for inclusion in the review.

Participants

Eligible participants were adults and children from families in high income countries as defined on the World Bank List of High Income Countries (worldbank.org.au). Low income countries were excluded as the availability of financial resources and the extent of malnutrition in these countries means that the issues are likely to be very different. The age range for inclusion of studies was children from birth through to 12 years (early childhood and primary school). The participants were ‘well’ children from the local population.

Socio-economic disadvantage was defined as families from areas that are described as disadvantaged (e.g. Low income area, ghetto, social housing projects); of low socio-economic status (e.g. low income as defined by the researchers); and disadvantaged minorities (eg. Indigenous peoples). Socio-economic disadvantage was not an inclusion/exclusion criterion, but was used to describe the impact of interventions on this population.

Interventions and specific comparisons to be made

Eligible interventions were those that aimed to improve nutritional intake undertaken for at least 12 weeks. In the RCTs, at least one group participated in a nutrition program and another group received a control intervention. Controls may have received no intervention, delayed intervention, or attention control. As an important aspect of school nutrition programs, school meals programs were included, updating the evidence from a 2007 review of school feeding programs [31]. Obesity treatment interventions for children were excluded as these programs have been reviewed recently [28, 32]. Obesity prevention interventions for children have also been reviewed [14, 15, 24] and were only included if the studies reported on nutritional outcomes in addition to changes in anthropometry.

Outcomes of interest

To be eligible for the review, a study had to report valid measures of at least one primary outcome.

### Primary outcomes

- Nutritional intake (measured by validated dietary assessment techniques, food purchasing, or biomarkers).
- Health status:
  - Any measure of physical health-Mortality, Morbidity, Hospital and Emergency department admissions.
  - Child growth and development outcomes- using standardised measures.
- Longer term effects following program completion (e.g. in the year(s) following to look at longer term outcomes)
- Adverse outcomes-Stigmatisation, dependency, decreased total family expenditure on food (including subsidy), increase in high fat/high sugar foods (including takeaway food), and obesity or excessive weight loss.

### Search methods

The following electronic databases were searched from 1980 to December 2014: Medline, Central (Cochrane)/DARE, Embase and CINAHL, with a search strategy that incorporated terms including both Medical Subject headings and keywords for: 1. Food, 2. Family-based programs and Institutional nutrition programs, 3. Nutrition and health outcomes. In addition, filters for high income countries and study design were applied. A Medline search strategy was developed and adapted to the other databases as required. The complete search strategy is attached as a Additional file 1. In addition, the reference lists of included studies were assessed to identify other eligible studies. Only articles published in English were included in the review.

### Data synthesis and analysis

The search results were downloaded into an Endnote library and titles and/or abstracts assessed by one of two authors for eligibility. The full-text manuscripts of those deemed potentially eligible were assessed by either author and any discrepancies resolved by consensus with the other author. Data of eligible studies were entered into a standard template and data entry checked after data extraction completed. The primary health and nutrition outcomes, any adverse outcomes, together with details of the intervention, including length of follow-up, demographics of participants and theoretical basis were extracted. The study authors were contacted to try to obtain additional data if necessary.

The interpretation of the results was facilitated by converting outcomes presented as a mean with a measure of variance to a Cohen’s d effect size estimate using an online calculator [33]. The impact of the interventions was compared using effect size (Cohen’s d) as a standard measure, which was interpreted as shown: Small 0.1–0.2, moderate 0.3–0.5, large > or = 0.6. Due to the heterogeneity of the interventions and study outcomes, a narrative synthesis of the results is presented [29].

### Quality appraisal

The included studies were assessed systematically for methodological quality and risk of bias using the Effective Public Health Practice Project (EPHPP) critical appraisal tool [34]. This tool assesses the risk of bias based on potential selection bias, study design, likelihood of confounding, blinding of outcome assessors and participants, appropriateness of data collection and completeness of follow-up. Studies were classified as high risk of potential bias if two or more of the above categories were assessed as weak (Weak), moderate risk of potential bias if one category was assessed as weak (Moderate) and low risk of potential bias if none of the above categories were assessed as weak (Strong).

## Results

**Fig. 1:**
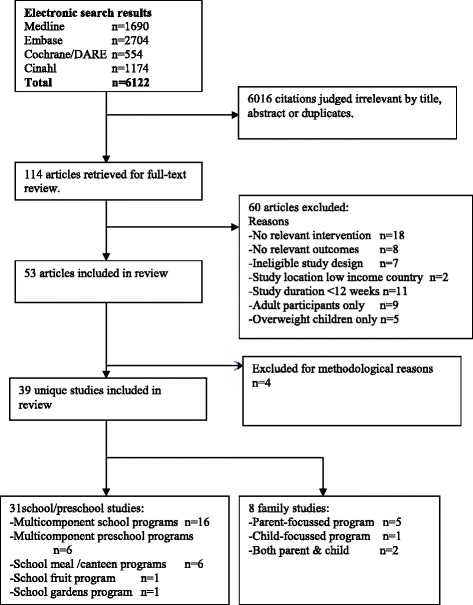
Flow chart of search results

The systematic literature search identified 6122 articles of which 786 were retrieved for abstract review. There were 43 studies that met the inclusion criteria (53 articles) and were included in the systematic review (see Fig. 1). A further 4 studies were excluded for inadequate reporting which prevented assessment of methods [35, 36] or outcomes [37], ineligible design [38] or no reporting of relevant outcomes [39]. Thus, this review reports on the results of 39 studies (Table 1).

**Table 1:** Characteristics of included studies

Study, Location	Setting, Disadvantaged population (if indicated)	Interventions	Participants age during intervention	Sample size	Sample size at follow-up	Duration of intervention	Follow-up duration post-intervention	Health & Nutrition Outcomes	Potential risk of bias rating^a^
Andersen 2014 [40] Damsgaard 2014 [83] OPUS school meal study Denmark	School	School meals (free)	Years 3 & 4 Mean age 10 years	834 no breakdown by I/C	663	6 month crossover trial	0	Dietary intake (self-report)	M
Anderson 2005 [50] Scotland	School	School multicomponent	Year 2 & Year 6 classes 6-7 years & 10-11 years	I=158 C=136	I=64 C=65	9 months	0	Dietary intake (self-report) Attitudes & knowledge of heathy eating	W
Baranowski 2000 [51] Gimme 5 USA	School	School multicomponent- comprehensive	Years 3-5	1864 no breakdown by I/C	1253	2 years	0	Dietary intake (self-report) F&V preferences, Self-efficacy	S
Baranowski 2003 [74] GEMS Fun, Food and Fitness USA	Family	Summer camp/ Internet F/U for family	8 years	I=19 C=16	I=17, C=14	3 months	<1mth	Dietary intake (self-report), BMI, PA levels (accelerometry, self-report)	M
Bayer 2009 [64] Tigerkids Germany	Preschool	Preschool -multicomponent	Preschool Mean age 6 years	I=1049 C=560, I2=1040 C2=565	I= 850, C=469; I2=872 C2=468	2 years	0	Dietary intake (Parent-report), BMI	S
Bere 2006a,2007, 2010 [45, 85, 88] Free School Fruit Norway	School	School-Free Fruit & multicomponent	Year 6/7 Mean age 11.3 years	577 no breakdown by IC	I=286 C= 231	2 years (only 1 year free fruit)	1 year, 3years	Dietary intake (self-report), F&V preferences	S
Bere 2006b [52] F&V Makes the Marks Norway	School	School- Paid fruit & multicomponent	Years 6/7 Mean age 11.3 years	450 no breakdown by I/C	I=190 C=179	1 year	1 year	Dietary intake (self-report), F&V accessibility, preferences, modelling	S
Breslin 2012 [70] Sport For Life Northern Ireland	School Low SES schools	School multicomponent	Year 3 8-9 years	I=209 C=207	?	3 months	1 month	Dietary intake (self-report), BMI, PA levels & Screen-time (accelerometry, self-report)	S
Caballero 2003 [53] Davis 2003 [94], Himes 2003 [95] Pathways study USA	School Native American community schools	School multicomponent	Year 3 Mean age 7.6 years	I=879 C=825	I=727, C= 682	3 years	0	Dietary intake (self-report, direct observation), BMI, PA levels (accelerometry, self-report)	S
Cameron 2014 [75] Campbell 2013 [96] Melbourne InFANT Australia	Family	Parent groups	Mother-infant pairs Infants 4-15 months	I=271 C=271	I= 241 C=239	18 months	0	Dietary intake (parent report), BMI, PA levels & Screen-time (accelerometry)	S
Christian 2014 [44] England	School	School gardening	Primary school ages 7-11	I=529 C=727	I=312 C=329	2 years	0	Dietary intake (observer & parent-report)	W
Cohen 2014 [54] CHANGE study USA	School Low income rural community	School multicomponent	Primary school Years 1-6	1302 no breakdown by I/C	I=251 C=181	2 years	0	Dietary intake (Assisted self-report)	M
Crepinsek 2006 [42] Universal Free School Breakfast Program USA	School	Free school breakfast program	Primary School Years 2-6	4358 no breakdown by I/C	I=2212 C=2066	3 years- Evaluation done at 12 months	0	Dietary intake (parent & self-report)	S
De Bock 2012 [65] Germany	Preschool	Preschool -multicomponent	Preschool Mean age 4.3 years	I=194 C=183	202 No breakdown by IC	6 months	6 months	Dietary intake (parent report), BMI	W
Epstein 2001 [20] USA	Family	Family - Parent sessions	30 families- 1 parent & 1 child Mean age 8.7 years	I=15 I2=15	27 No breakdown by IC	6 months	6 months	Dietary intake (parent & self-report), BMI	M
Evans 2013 [55] Project Tomato England	School	School multicomponent	Year 3 (7-8yrs)	I=530 C=550	I=331 C=347	10 months	10months	Dietary intake (observer & parent-report)	S
Greening 2011 [56] TEAM Mississippi Project USA	School Low SES rural community	School/Family	6-10 years Mean age 8.3 years	I=224 C=283	I=204 C=246	8 months	0	Dietary intake (parent-report), Fitness & PA levels	S
Hardy 2010 [73] Munch & Move Australia	Preschool	Preschool -multicomponent	Mean age 4.5yrs	I=263 C=167	I=218 C=141	5 months	0	Lunch foods (Measurement), Movement skills	M
Hendrie 2011 [76] Hendrie 2013 [97] Australia	Family	Family - Parent sessions	4-13 year old children Mean age 8.6yrs	I=76 C=69	I=76 C=64	3 months	3 months	Dietary intake (parent & self-report), BMI, Plasma lipids	M
Hendy 2011 [46] Kid’s Choice Program USA	School	School rewards program (Lunch)	Years 1-4	457 Both groups	382 both groups	3 months	0	Dietary intake (observation), BMI, PA levels (Pedometery)	S
Hoffman 2010 [57] USA	School Low SES urban community	School multicomponent	Kinder, Year 1 Mean age 6.2 years	I=149 C=148	I=113 C=97	6 months	1 year	Dietary intake (observation), BMI, F&V preferences	W
Hopper 1996 [69] USA	School	School multicomponent	Year 2 & 4 Mean age 8.9 years	I=48 C=49	I=48 C=49	12 weeks	0	Dietary intake (Assisted self & parent-report),BMI	M
Horne 2009 [58] Food Dudes Ireland	School	School multicomponent	4-11 years	I=228 C=207	?	12 months	0	Dietary intake (observation)	M
Kristjansdottir 2010 [59] Iceland	School	School multicomponent	7-9 years	265 No breakdown by I/C	I=58 C=48	2 years	0	Dietary intake (Parent report & measurement)	W
Luepker 1996 [60], Dwyer 2002 [84] CATCH USA	School	School food service changes, curriculum and home program	Year 3 initially 8 to 11 years	5106	4019 (1532 at 3 years post intervention)	3 years	3 years	BP, BMI/skin fold thickness, serum lipids and Apolipoprotein B, dietary intake (self-report), PA/screen time (self-report), fitness (9 minute run)	S
Llargues 2011 [61] Avall Study Spain	School	School based curriculum multicomponent, plus home	Year 1 5-7 years	598, I=272 C=237	I=272, C=236	2 years	0	BMI, dietary intake (parent-report), PA/screen time (parent-report)	S
Moore 2007, 2014 [41]^,^ [98] Murphy 2011 [82] Welsh Primary School Free Breakfast Initiative Wales	School	Free school breakfast program	9 to 11 years	RCT trial with repeated X sectional design	1975 had at least baseline and one follow up	12months	0	Dietary intake (Self-report), Classroom cognitive tests, Attitudes towards breakfast (Self-report), Strengths & Difficulties questionnaire (teacher-report)	W
Muth 2008 [62] IMPACT USA	School	School curriculum based	Year 4 Mean age 9.9 years	I=38 C=37	I=37 C=36	3 months plus student leader training	0	Dietary intake (Self -report), PA/sedentary behaviour/screen time (Self -report)	S
Natale 2013 [66], [99] Healthy Caregivers, Healthy children USA	Preschool	Pre-school multicomponent, environmental and family	2 to 5 years	I=726 C=379	I=238 C=69	2 years	0	Dietary intake (parent-report), sedentary behaviour (parent-report)	W
Natale 2014 [67] Healthy Inside-Healthy Outside (HI-HO) USA	Preschool	Pre-school multicomponent plus home	2 to 5 years	I=238, C=69 dyads	185 dyads	6 months	6 months	Dietary intake (parent-report), BMI, PA levels and screen time (parent-report)	M
Ni Mhurchu 2011 [43] Free school breakfast New Zealand	School	Free school breakfast program	Primary school children Mean age 9.4 years	424	375	1 year for longest	0	Breakfast intake/hunger (self-report), food security (family-report) Academic achievement, school attendance (records), Strengths & Difficulties (teacher-report)	M
Olvera 2008, 2010 [77, 81] Bounce program USA	Family Latino mothers/ daughters	Community-based PA and nutrition intervention	Years 3 to 6	46 dyads, I=26 C=20	I=18 C=17	12 weeks	0	Dietary intake (self & parent report), BMI, PA (accelerometer, shuttle run test) exercise heart rate, peak oxygen consumption	M
Perrikou 2013 [72] Cyprus	School	Teacher modelling (EXPO), curriculum (EDUC) two IV streams	9 years	I1=68, I2=79, C=71	I1=59, I2=67, C=58	29 weeks	1 year	Dietary intake (observation)	M
Perry 2004 [47] Cafeteria Power Plus Project USA	School	Cafeteria-based intervention	Years 1 & 3	1668, no breakdown by I/C	1168	2 years	0	Dietary intake (parent-report), BMI	M
Raitakari 2005 [49], Kaitosaari 2006 [48] Special Turku Coronary Risk Factor Intervention Project for Children (STRIP) Finland	Family	Family-based low saturated fat diet/nutrition intervention	Infants 7 months old at commencement	I=540 C=522 (families as unit)	I=179 C=190	Ongoing FU visits	9 years & 11 years	Dietary intake (self & parent report), PA (self-report), endothelial function (brachial artery ultrasound), BP, BMI, lipids, glucose, insulin, HOMA-IR, Apolipoprotein A, Apolipoprotein B	W
Rush, 2012 [63] Project Energize New Zealand	School	School based nutrition and PA change agent mediated	Primary school children Years 1 -6	I=692 C=660	1352 (~30% lost to follow up, analysis restricted to matched groups)	2 years	0	BP, BMI, % body fat (bioelectrical impedance)	M
Stolley 1997 [78] USA	Family Low income African American families	Community-based PA and nutrition IV	7-12 year olds	Pairs I=32, C=33	I=23 daughters & 20 mums C=27 daughters &18 mums	12 weeks	0	BMI, dietary intake (self-report)	M
Tabak 2012 [79] Family Ties to Health Program USA	Family	Family - Phone calls and newsletters	Preschool-aged children 2-5 years	I=25 C=23	I=22 C=21	4 months	0	Dietary intake (parent report)	W
Vereecken 2009 [68] Beastly Healthy at School Belgium	Preschool	Pre-school multicomponent	Preschool children 2.5 to 5 years	I=742 kids C=480	I=618 kids C=445	12 weeks	6 months	Dietary intake (observation and parent-report)	S

^a^ *S* Strong (Low risk of bias), *M* Moderate (Moderate risk of bias), *W* Weak (High risk of bias)

### Description and scope of the included studies

The included studies were all RCTs. Almost half the studies (*n* = 18) were conducted the United States with 16 studies in a range of European countries, including five in the United Kingdom, as well as three studies in Australia and two in New Zealand. The dates of publication ranged from 1996 to 2014 however, 31 of the studies were published in the last 10 years.

The majority of the studies (82%) were undertaken in schools (*n* = 25) or preschools (*n* = 6) with the most common approach incorporating multiple components to promote healthy eating and/or other aspects of healthy lifestyle particularly increased physical activity (Table 1). These multiple component nutrition programs included combinations of class curriculum activities, school food service modifications, home activities, enhanced physical education/activities and strategies to engage parents/families (see Table 2). There were also four studies of school meal programs [40–43], one school gardening study [44], a school fruit program study [45] and two school canteen studies [46, 47]. The duration of the studies ranged from 3 months to the ongoing STRIP studies [48, 49] which reported follow-up of children recruited as infants at 9 and 11 years. Twenty-three studies (59%) were between 6 months and 2 years in duration (Table 1).

**Table 2:** Approaches used in various combinations in the multi-component school/ preschool programs and family-based programs

School/Preschool	
• Fruit and or vegetable snacks (Free or Paid)	
• School lunch program	
• School garden	
• Improvements in school meals/tuckshop/canteen facilities	
• School breakfast program	
• Nutrition education- Classroom	
• Meal preparation sessions child (and/or parent)	
• Physical activity sessions	
• Change agent to support healthy nutrition/physical activity	
• School food policy changes	
• Observation/Rewards eating healthy foods at lunch/in class	
• School-wide promotion messages	
• Multimedia activities promoting healthy lifestyles	
• Teacher modelling healthy eating	
• Homework activities/Newsletter	
Family	
• Individual and/or Group Nutrition education	
• DVDs/Newsletters/Workbooks	
• Non-residential camp	
• Practical nutrition and/or physical activity sessions	
• Internet education/activities	
• Phone call from program staff	

Fourteen of the 25 school studies included efforts to involve families [50–63], however, the uptake of this was limited in most studies where it was assessed [51–53, 55, 60]. There was only one school study predominantly focussed on family activities, however, it was less intensive as it involved monthly nutrition or physical activity challenges [56]. Five of the six preschool studies involved parent-focussed activities [64–68], with three of these including parents in nutrition education sessions [65–67].

The participants in the primary school based programs were predominantly older children aged 8–12 with many of the studies at multiple schools involving hundreds of students. There were two smaller scale programs at single schools with 60–100 participants [62, 69]. There were three particularly large studies- a trial of universal free school breakfast program in the USA (compared with the existing means-tested school breakfast program) assessing 4358 students (153 schools) [42], an evaluation of a free school breakfast program in Wales involving 4350 students at 111 schools (1975 with follow-up at all time-points) [41], and the Child and Adolescent Trial for Cardiovascular Health (CATCH), a multi-component school program in the USA with 5106 students (96 schools) at baseline [60]. The majority of the school and preschool studies involved populations that were representative of the community and thus included children from families with varying socio-economic status (SES)/educational attainment. Six studies included predominantly disadvantaged children, either by selecting schools fulfilling criteria for low SES [41, 57, 70], an intervention for Native American children [53], or by undertaking the study in low income rural communities [54, 56].

The majority of the studies in schools and preschools involved a mixture of support for school staff, either teachers or cafeteria staff, and/or research staff to deliver enhanced nutrition education including opportunities for practical food experiences [44, 47, 50–53, 57, 59, 61, 65–72]. These were usually at one or more geographically clustered schools. In addition, two studies had a major focus on teachers and/or parents as role models of healthy eating [66, 72], while another study involved high school students as facilitators of the nutrition education [62]. There were six studies which instituted large scale programs- either developed directly with the input of the education or health department [58, 63, 73], or instituted with low-cost resources or requiring low intensity input by school staff that was consistent with the local school curriculum [55, 60, 64]. The three publicly funded programs included Food Dudes in United Kingdom primary schools [58], Project Energise in primary schools in Waikato, New Zealand [63] and Munch and Move in preschools in New South Wales, Australia [73]. In addition, there were studies evaluating publicly funded national school meals programs- breakfast programs in the USA [42] and Wales [41] and a school fruit program in Norway [45]. There were also smaller research trials of a school breakfast program in New Zealand [43] and a school lunch program in Denmark [40].

There were only eight family-based studies (Table 1) and these predominantly targeted the parents or parents together with their children [20, 49, 74–79]. Four of the five family-based studies aimed at parents only involved monthly (or less frequent) nutrition education for individuals or groups together with individual assessment [49, 75, 76, 79]. However, the two studies involving parents with their children incorporated practical nutrition or physical activity sessions one to three times a week [77, 78]. The only study to directly target children involved a 4 week summer camp program in the USA with internet follow-up for the children and their parents [74]. These family-based studies had relatively small numbers of participants except for an Australian study involving parent groups for those with infants [75] and the Special Turku Coronary Risk Factor Intervention Project for Children (STRIP) [49]. The recruitment strategies used in these studies meant that unless a disadvantaged population was explicitly targeted [77, 78], then participants were predominantly not disadvantaged [74–76, 79].

### Overall methodological assessment

Of the 39 RCTs, 15 (38%) were assessed to be at low risk of bias, 15 were assessed to have a moderate risk of bias and 9 to have a high risk of bias (Table 1). Underlying theories and/or clear rationale for interventions were common among the included studies. The development of 26 of the studies were based on one or more specific theories of behaviour most commonly social cognitive theory (*n* = 13) and social learning theory (*n* = 7). In addition, the five school meals programs were developed with a clear rationale. There was no clear relationship between a theoretical basis or rationale and effectiveness of the intervention.

Nutritional intake was assessed using standardised self-reported (or parent-report) measures in 29 of the 38 studies which reported these data (76%) with two of these incorporating assistance by research staff and one using measured self-reporting. Five studies (13%) used measurement/observation by research staff and four (11%) used a combination of self-report and observation. In addition, most of the studies reported standard categories of macro- and micronutrient intake alongside other outcomes, which increases the likelihood of finding statistically significant differences by chance alone due to multiple comparisons [80]. Finally, only 12 studies (31%) reported on follow-up at a time after the intervention, and of these only three reported long-term follow-up greater than 1 year (Table 1).

Despite these potential methodological limitations, the consistent results from these RCTs and the observation that most appeared adequately powered to detect relevant improvements in health outcomes for individuals and populations provides support for the findings of this review.

### Outcomes

The focus of this review and hence the most frequent outcome reported was dietary intake, with all but one study reporting on dietary intake through at least one measure. The most commonly reported outcomes were fruit and vegetable intake and fat intake. The intake of energy-dense nutrient-poor foods was another focus analysed in fewer studies. These are reported in detail below.

Many of these programs also aimed to promote healthy lifestyles. Hence in addition to dietary intake, physical activity levels and/or sedentary behaviour was reported by 15 studies (12 school studies and three family studies), screen-time in six studies and body mass index was reported by 19 studies (12 school studies and all seven family studies). Analysis of these outcomes was not undertaken. Other biomedical outcomes were reported in only four studies, which were generally the longer-term studies and/or more intensive studies aimed at reduction of fat intake and adiposity. The determinants of improved dietary intake were reported by a limited number of studies- attitudes and knowledge in five school studies, self-efficacy in two studies and F&V access in one study. Adverse effects were not reported in these studies.

There were 93 effect size estimates extracted or calculated in the 39 included studies. 31 (33%) of these effect sizes (Cohen’s d) were greater than 0.2 and had a 95% confidence interval that did not include the null value. However, there were also five studies which reported no impact on intake of energy-dense, nutrient-poor foods and three studies which reported no effect on at least one biomedical outcome, and no effect size estimates were calculated. The 31 positive effects were all increasing F&V intake or reducing fat intake; however there was no obvious relationship between study components and effectiveness. The impact of involving parents in school/preschool studies was only systematically assessed in the CATCH study [60], which did not find a difference in outcomes between the school-based intervention with or without parental involvement.

### Impact on fruit and vegetable (F&V) intake

Twenty-five studies reported on F&V intake (Table 3). Of these studies, five reported a null effect on fruit and/or vegetable intake [44, 50, 52, 55, 75]. There were eleven studies which found a null to small effect on F&V intake [45, 47, 51, 54, 57, 64, 68, 70, 73, 79, 81]. Five studies reported a moderate effect on F&V intake [46, 58, 62, 65, 69] and four studies which found a large effect on F&/or V intake [20, 59, 72, 74]. In addition, two studies which reported a null to small effect on vegetable or overall F&V intake, also reported a moderate to large effect on fruit intake [50, 57]. Ten of the 25 studies were assessed to be at low risk of bias (Table 1) with eight of these reporting null to small effect on F&V intake.

**Table 3:** Impact of nutrition interventions on children’s fruit and vegetable intake

Study	F&V results^a^ (95% CI)	Outcome method	Direction of assoc. Intervention vs control
Family studies			
Baranowski 2003	Fruit & Veg (including juice) intake d = 1.3 (0.55, 2.11)	24-h recall ×2	+
Cameron 2014	Fruit intake d = 0.06 Veg intake d = 0.05	24-h recall ×3	–
Epstein 2001	F&V intake d = 1.05 (0.23–1.87) (↑ F&V group cf. ↓Fat/Sugar group)	FFQ	+/−
Olvera 2008, 2010	F&V d = 0.34	SPAN questionnaire	+
Tabak 2012	V intake d = 0.24 (−0.35, 0.84)	FFQ	–
Preschool/School studies with no parent component			
Bere 2006a	F&V intake (1 yr) d = 0.21 (0.04, 0.39) F&V intake (2 yrs) d = 0.19 (0.02, 0.37)	24-h recall	+
Breslin 2012	Veg intake d = 0.17 (−0.03, 0.36) Fruit intake d = 0.0 (−0.19, 0.19)	FFQ	–
Christian 2014	F&V intake d = −0.2 (−0.3, 0.0) Veg intake d = −0.1 (−0.2, 0.1) Fruit intake d = −0.1 (−0.3, 0.0)	24-h recall	–
Hardy 2010	Fruit in lunch d = −0.11 (−0.40, 0.18) Veg in lunch d = 0.20 (−0.09, 0.48)	Lunchbox audit	–
Hendy 2011	F or V eaten first d = 0.61 (0.36, 0.81)	Direct observation	+
Perrikou 2013	Fruit intake d = 1.43 (1.05, 1.79) Exposure d = 1.41 (1.05, 1.77) Education at 1 year	2 day food record (parent)	+
Perry 2004	Fruit intake d = 0.09 Fruit (no juice) d = 0.12	Direct observation	+
Preschool/School studies with parent component			
Anderson 2005	Fruit intake d = 0.48 (0.13, 0.83) F&V d = 0.04 (−0.30, 0.39)	3 day food record (self-report)	+/−
Baranowski 2000	F&V intake Mean difference 0.2 serves/day (1 yr), 0.2 Serves/day (2 yrs)	7 day food record	+
Bayer 2009	Fruit intake d = 0.14, Veg. intake d = 0.13	FFQ	+
Bere 2006b	F&V intake (1 yr) d = 0.03(−0.17, 0.23) F&V intake (2 yrs) d = −0.1(−0.29, 0.12)	24-h recall	–
Cohen 2014	F&V intake/1000 kcal d = 0.1 F intake/1000 kcal d = 0.1 V intake/1000 kcal d = 0.1	24-h recall/FFQ	–
De Bock 2012	Change in Fruit intake d = 0.38 (0.18, 0.59) Change in Veg intake 0.33 (0.12, 0.53)	Short questions	+
Evans 2012	F&V intake d = 0.01 (−0.14, 0.16)	24-h recall	–
Hoffman 2010	Fruit intake 1 yr. d = 0.86, 2 yrs. d = 0.55, Veg intake 1 yr. d = 0.34, 2 yrs. No difference (at school)	Direct measurement	+/−
Hopper 1996	Change in F&V serves d = 0.40 (0.00, 0.80) (at school)	Direct observation & measurement	+
Horne 2009	F&V consumed at lunch at 1 yr. d = 0.35 (0.16, 0.54)	Direct observation & measurement	+
Kristjansdottir 2010	F&V intake d = 0.92 (0.52, 1.32)	3 day weighed food diary	+
Muth 2008	F&V Mean difference + 0.85 serves/day	SPAN questionnaire (child)	+
Vereecken 2009	Fruit intake d = 0.19 (0.00, 0.38) Veg intake d = 0.08 (−0.11, 0.27) Fruit intake (at school) d = 0.10 (−0.02, 0.22)	FFQ (parent) Audit (teacher)	+/−

^a^d = Cohen’s d where data available otherwise mean difference presented

Overall, there is evidence that both school-based studies and family-based studies, if designed and implemented well, have a positive impact on F&V intake, particularly fruit. These successful programs incorporated engaging and innovative strategies including multimedia programs to motivate the children. This impact was demonstrated for the duration of the intervention and for up to 12 months post-intervention follow-up. For example, the one study of a school garden program found no impact on F&V intake overall, but did report a significant increase in F&V intake in students at schools where a successful school garden was achieved [44]. However, the majority of the studies had a null or small effect on F&V intake.

There were two family-based studies and five school studies that reported a medium or large effect on F&V intake and were at low or moderate risk of bias. Five of these studies were based on social cognitive theory or social learning theory and involved high intensity interventions and/or innovative strategies to engage children. The family-based studies included the GEMS study involving 8 year-old girls at a 4 week summer camp in the USA with weekly internet sessions for girls and their parents afterwards for 8 weeks [74]. This study incorporated activities to promote F&V intake and physical activity into the regular camp program. The other family based study involved obese parents and their non-obese children in a comprehensive weight-control program for the parents comparing the impact of promoting F&V or low fat/sugar diets [20]. Both of these studies were delivered with high fidelity and had high follow-up albeit in small sample size. The five school studies included one which compared exposure to a teacher modelling eating fruit (or other healthy snacks) daily or healthy lifestyle curriculum with a control group [72]. Both of the intervention groups increased fruit intake at the end of the 1 year intervention. However, only the exposure group maintained this increased fruit intake 1 year later. There was also a lunch rewards program with observation 3 days/week [46]. Another school curriculum based program was of low intensity but involved high school students to deliver the program to younger children [62]. All three of these were delivered with high fidelity and achieved high follow-up. There were two other school programs, one involving daily videos and rewards together with F&V provision [58] and the other involving four physical activity and two nutrition education sessions weekly [69]. However, the fidelity of these two interventions and the follow-up achieved was not clear. Three of these five school studies [58, 62, 69] involved parents in some way while the other two had no parental involvement [46, 72], however there was no systematic assessment of the impact of involving parents in any of these studies (Table 3).

### Impact on fat intake

Fifteen studies reported fat outcomes (Table 4). Of these studies, six had a null effect [40, 44, 50, 54, 55, 59], four had a null to small effect [42, 53, 60, 69], three had a moderate effect [56, 78, 81] and two studies a large effect on dietary fat intake [48, 76]. Five of the 15 studies were assessed to have a low risk of bias (Table 1) with one of these reporting a null effect, three a small effect and one a moderate effect on fat intake. Overall, there is some evidence of benefit for studies that specifically target fat intake and are set in the home/parent-based intervention, although the majority of the studies had a null or very small effect.

**Table 4:** Impact of nutrition interventions on fat intake of children

Study	Fat results (Cohens’ d or mean difference^a^ (95% CI)	Outcome method	Direction of assoc. Intervention vs control
Family studies			
Hendrie 2011	Total fat Mean diff = −10.9 g/day (−19.3, −2.5), Saturated fat Mean diff = −8.1 g/day (−11.9 to −4.3)	24 h recall ×3	+
Kaitosaari 2006 STRIP study	Total fat Boys d = −0.24 (−0.68, 0.20); Girls d = −0.78 (−1.22,-0.34) Saturated fat Boys d = −0.79 (−1.22,-0.34); Girls d = −1.17 (−1.63, −0.71) Polyunsaturated fat Boys d = 0.55 (0.10, 0.99); Girls d = 0.62 (0.19, 1.06)	4 day food record	+
Raitakari 2005	Saturated fat Girls age 11 d = −0.46 (−0.76, −0.17) Boys d = −0.95 (−1.25, −0.64)	4 day food record	+
Olvera 2008, 2010	Reduced intake of high fat foods d = 0.39 (−0.28, 1.06)	FFQ	+
Stolley 1997	Saturated fat d = 0.39 (−0.17, 0.95) Fat % of Total energy d = 0.54 (−0.02, 1.11)	FFQ	+
School studies with no parent component			
Andersen 2014	Total fat d = 0.00 (−0.11,0.10), Saturated fat d = 0.00 (−0.11, 0.10), Monounsaturated fat d = 0.00 (−0.11,0.10) Polyunsaturated fat d = 0.00 (−0.10,0.10) Trans fatty acid d = 0.00 (−0.10,0.10)	7 day food diary	–
Christian 2014	Total fat intake d = 0.02 (−0.13, 0.18)	24 h recall	–
Crepinsek 2006	Total fat d = −0.11(−0.17, −0.04) Saturated fat Mean diff = −0.20% (% of total energy)	24 h recall	+
School studies with parent component			
Anderson 2005	Fat as % energy Mean diff = −0.1%	3 day food diary	–
Caballero 2003 Pathways study	Total Fat Mean diff = −2.5% (−3.9,-1.1) Total fat at lunch Mean diff = −4.2% (−7.1,-1.3) (% of total energy)	Direct observation, 24 h recall	+
Cohen 2014	Energy from saturated fat d = −0.02 (−0.21, 0.17)	24 h recall, Modified FFQ	–
Evans 2012	Total fat Mean diff = 1.2 g/day (−2.8,5.1), Saturated fat Mean diff = 0.0 g/day (−1.5,1.5)	24 h recall	–
Greening 2011	Reduced dietary fat d = 0.33 (0.15, 0.52)	Child dietary fat questionnaire	+
Hopper 1996	Saturated fat Mean diff = −0.63 g/day (*p* > 0.05)	24 h recall	+
Kristjansdottir 2010	Total fat (g/day) d = 0.18 (−0.2,0.56) Saturated Fat d = 0.04 (−0.34, 0.42) Monounsaturated fat d = 0.35 (−0.04, 0.73) PUFA d = 0.03 (−0.35, 0.41)	3 day weighed food record	+/−
Leupker 1996	Fat as % energy d=-0.23 (-0.35, -0.11) Monounsaturated fat d= -0.15 (-0.26, 0.03) Saturated fat d=-0.20 (-0.32, -0.09) Polyunsaturated fat d=- 0.16, (-0.28, 0.045) (All % Total energy)	24 h recall	+

^a^d = Cohen’s d where data available otherwise mean difference presented

The two studies with a large effect had reduction in fat as a main aim of the study, whereas the majority of the other studies focussed on fruit and vegetable intake. Hendrie et al [76] targeted parents of school-aged children to promote low fat dairy products. This study involved a low intensity intervention in a relatively small sample size; however it was delivered with high fidelity, based on social learning theory and a high follow-up was achieved. In addition to the reduction in fat intake, there was a small change in LDL cholesterol, but no change in BMI. The STRIP study [48, 49] is an ongoing, relatively low intensity program in which families with infants were recruited to attend twice yearly nutrition education and assessment promoting a low saturated fat diet, predominately via parents. This study also had high fidelity, but was not based on any explicit theory. These STRIP studies reported on the follow-up of a small sub-sample, aged 9–11 years at follow up. In addition to these two family-based studies, four of the eight school studies involving parents [53, 56, 60, 69] (all assessed to have a low risk of bias) reported smaller reductions in fat intake of children, although the only study which tested the impact of parental involvement systematically reported no additional benefit compared to no parental component [60] (Table 4).

### Impact on energy dense, nutrient poor (EDNP) foods intake

There were nine studies which reported intake of EDNP foods. Of these, three found no effect [62, 66, 73], two studies reported a small effect for one outcome measure but not the other related to EDNP foods [64, 82], two showed a moderate effect (although findings not statistically significant) [20, 81]. and two studies had limited reporting of results [67, 70]. Overall, the studies demonstrated limited evidence for benefits in reducing EDNP food intake.

The studies reporting a moderate effect were family-based studies. Epstein et al. recruited obese parents, with non-obese children, to a weight control program, with a reduction in EDNP foods among the children (d = −0.51 95% CI -1.30, 0.27) for those in the arm that focused on reducing intake of high fat/sugar foods (compared with the F&V arm) [20]. This study had a small sample size, moderate intensity over 6 months and then 6 months post intervention follow-up and was at moderate risk of bias. The Bounce program involved healthy lifestyle activities after school for mother-daughter pairs, with reductions in high fat foods (Cohen’s d = 0.40 *p* = 0.26) and sweetened beverages (Cohen’s d = 0.36, *p* = 0.31) among girls in the intervention group [81]. This was also a small study with high intensity over 3 months and a moderate risk of bias. Given the nature of these studies, the results should be interpreted cautiously.

### Other health outcomes

Five of the studies reported biomedical outcomes, although as the duration/follow-up of the studies was < 3 years, except for the STRIP study, the focus was on proxy measures. There was a null effect on blood pressure and lipids in the STRIP and CATCH studies [48, 60]. The STRIP studies found a small to moderate effect on reducing insulin resistance in 9 year old children [Cohen’s d = −0.16 (95% CI -0.58, 0.27) in girls and −0.58 (95% CI -1.02, −0.13) in boys] and null to small effects on lipids [48]. In a later follow-up, in 11 year olds, there was a small to moderate effect on improving endothelial function [Cohen’s d = 0.11 (95% CI -0.18, 0.39) in girls and 0.34 (95% CI 0.05,0.63) in boys] with reduced total and LDL cholesterol in boys only [49]. There was decreased blood pressure (BP) in the 10–12 year old children [systolic BP mean difference = −0.23 (95% CI -0.43,-0.02); diastolic BP mean difference = −0.14 (95% CI -0.30, 0.04)] but not the 5–7 year old children in Project Energise-a large school-based healthy lifestyle program in New Zealand [63]. Project Energise involved a change agent to champion the promotion of healthy nutrition and increased physical activity in curricular and extra-curricular activities in primary schools. The study also found small decreases in adiposity in 5–7 year old children only. In addition to the reductions in fat intake, Hendrie et al. found reduced LDL cholesterol mean difference = −0.15 mmol/L (95% CI -0.30, −0.01, Cohen’s d = 0.52) and total cholesterol mean difference = −0.12 (95% CI -0.28, 0.05) but no changes in other plasma lipids 3 months after the program [76]. In the school lunch study in Denmark, Damsgaard et al. found no change in the metabolic syndrome score with reductions in mean blood pressure, total cholesterol, TAG and insulin resistance offset by increased waist circumference and reduced HDL cholesterol [83]. Of these five studies, only the CATCH study was assessed to have a low risk of bias. Thus, the limited evidence of improvements in cardiovascular disease risk factors, should also be interpreted cautiously.

### Dietary and health outcomes and socio-economic status/disadvantaged populations

The five school-based studies and two family based studies which were targeted at disadvantaged populations (Table 1) did not clearly demonstrate that these programs were more or less effective in these populations compared to the overall assessments described above. Four of the studies reported on F&V intake with one reporting a large effect [57], one a moderate effect [81] and two a small effect [54, 70] (Table 3). Four of the five studies which reported on fat intake in these populations showed moderate effect [53, 56, 78, 81] and one showed no effect [54] (Table 4). The Bounce study, described above, involved disadvantaged African-American mother-daughter pairs in an urban setting and reported reduced fat intake, increased F&V and reductions in EDNP food, as noted previously [81]. This is consistent with the sub-group analysis reported in the 3 year follow-up CATCH study, which showed that overall dietary intake after this program in rural USA was the same in African-American and Hispanic- American children as in white children [84].

### Sustainability of nutrition and health outcomes

There were four studies of three or more years duration included in the review [42, 49, 53, 60]. Three were school-based programs, including two multi-component programs- the Pathways program which aimed to reduce adiposity in American Indian school children and the CATCH program which aimed to reduce risk factors for cardiovascular disease in primary school students (Table 1). Both reported small to moderate effect on reducing total fat and/or saturated fat intake after 3 years: Pathways Total fat intake mean difference-2.5% and CATCH Total fat intake mean difference-2% (Table 4). The other school-based program was the Universal School Breakfast Pilot Program in the USA [42]. This program operated successfully for 3 years; however, follow-up dietary intake data were only collected at 12 months. This showed that intake of a nutritionally substantive breakfast increased from 76% to 80% in intervention schools, but that breakfast skipping was 4% in both intervention and control schools. The other long-term study was the family-based STRIP study in Finland. The children participating in this program had lower intake of total and saturated fat at 9 years and saturated fat at 11 years than control children, although results varied by gender (Table 4).

Long-term follow-up of programs (> 1 year post-intervention) to assess the ongoing impact was reported by two studies in addition to the ongoing STRIP study described above. A 3 year follow-up of the CATCH study reported that these children still had significantly lower intake of total and saturated fat compared to controls although overall dietary intake was not significantly different between the two groups (based on the Healthy Eating Index) [84]. In a 3 year follow-up of the Free School Fruit program in Norway, increases in F&V intake, noted at the conclusion of the program and 1 year after the program, persisted although reduced from the conclusion of the program [85]. It was estimated that boys ate an extra 0.38 serves of F&V/day and girls 0.44 serves of F&V/day as a result of prior participation in this school fruit program compared to an extra 0.6 serves/day at the conclusion and 0.5 serves/day at 1 year [45]. Thus, there is consistent although limited evidence that ongoing multicomponent nutrition programs can improve dietary patterns sufficiently to impact on population health (if sustained) and that these effects persist, although attenuated for up to 3 years.

## Discussion

The majority of the 39 RCTs included in this review were in schools or preschools with only eight undertaken in family settings. There were substantial (and statistically significant) improvements in dietary intake in only 31% of outcomes assessed- all related to increased F&V or decreased fat intake. There were a small number of effective family-based programs which delivered simple dietary information to empower parents and/or engaged parents and their children directly with ongoing follow-up in person or using the internet. Overall, however, there is insufficient evidence to determine the impact of involving parents in school/preschool nutrition programs. Only a small number of longer term child nutrition programs have been undertaken, largely in schools. Such programs rely on ongoing availability of resources which may limit the sustainability of both the program and any health outcomes. Further, sustainability in long term programs is dependent on ensuring the engagement of participants in an effective program.

The findings do indicate the importance of aspects of social context to healthy eating. The two studies which reported a large decrease in fat intake (both directed at parents) indicate that the promotion of simple dietary information which is well understood and engages parents is effective and enables them to have a major impact on their children’s dietary intake [48, 76]. Similarly, school nutrition programs can improve the intake of healthy foods, particularly fruit. Based on successful outcomes observed in this review, the most effective strategies included the use of innovative strategies to engage and motivate the children including rewards, cartoon characters promoting healthy foods, modelling by teachers and the use of older peer educators. A theoretical framework, particularly the use of social cognitive theory or social learning theory, also appeared to support positive impacts of these programs. None of these features were sufficient to achieve positive outcomes; rather it supports that the effective implementation of an innovative and well-designed intervention is more likely to improve dietary intake.

In a 2010 review, Hingle et al. [27] concluded that there was insufficient evidence of the impact of parental involvement in dietary interventions to improve children’s dietary intake. This is consistent with our finding that although parental/family involvement was an element of most school/preschool studies, it was not central to most interventions or consistently implemented. Hingle et al. did find that more direct approaches to engage parents were more likely to have a positive impact on children’s diet. The positive impacts demonstrated in the family studies in this review provide further evidence to support this finding. The use of social cognitive theory or social learning theory to enhance the effectiveness of nutrition interventions has been previously identified [27, 86]. Interestingly, Segal et al [87] have highlighted that it is the extent to which the theory is consistent with the needs of the target population and the program components are consistent with the nominated theory which may enhance effectiveness. Thus, identifying and explicitly assessing elements of social cognitive theory (or other relevant theory) will enhance the understanding of how to design and implement an effective nutrition program.

The sustainability of the dietary improvements of successful nutrition programs is vital to achieving long-term health benefits. The three studies [49, 84, 88] reporting on longer term follow-up reinforced that simple dietary interventions, such as promoting a lower fat diet to families (STRIP study) or a school fruit program- can produce sustained improvements in dietary intake. Ongoing large scale nutrition programs in schools/preschools are easier to implement and maintain provided funding and support is available. This was demonstrated by three school studies of 3 years duration including the Pathways program in Native American schools [53, 60], and the US free school breakfast program [42]. There is also the large scale implementation of other preschool and school-based programs, such as Food Dudes in the UK [89], Project Energise in New Zealand [63] and Munch and Move in Australia [73]. It is relevant to consider how practical it is to implement complex nutrition programs in other settings. Published articles cannot include the details of educational resources or activities undertaken and there are resource implications of making this material available online. It is also difficult to convey or replicate the interpersonal interactions within program activities that may contribute to the intervention’s effectiveness. The Food Dudes program demonstrates the sustained commitment and resources required to implement a successful nutrition intervention in other locations [89, 90].

From a population perspective, the sustainability of nutrition programs must also be weighed against the opportunity cost implicit in funding such an ongoing program. Overall, these programs have only a modest impact on dietary intake, and alone are unlikely to mitigate the many changes within contemporary society which have undermined healthy dietary intake and lifestyles. Other strategies which help to create an environment which supports people’s health and nutrition, including restricting junk food advertising or enhancing the availability of healthier food choices, need to be compared with the impact of both school and family nutrition programs. Considered assessment of these strategies, including economic analysis, will help to develop a range of effective programs that together can support healthy eating in the population.

The potential to improve dietary intake is relevant to most of the population. This review found that school and community nutrition programs undertaken in disadvantaged communities were as effective as interventions in other settings and thus should impact dietary intake sufficiently to improve health outcomes in these populations. However, there was limited reporting of results by socio-economic status (SES) in the larger school studies, unless it was relevant to the rationale for the intervention, such as school meal programs. It was also observed that family-based programs recruited predominantly families with above average SES, unless undertaken explicitly targeting a disadvantaged community. Thus, the evidence suggests that child nutrition programs are beneficial in all children irrespective of SES, although it is important to consider strategies to maximise disadvantaged families’ participation in effective family-based nutrition programs.

The most important limitation in this review was the validity and reliability of the self-reported dietary intake data. Although the studies used standardised methodologies predominantly 24-h recalls and FFQs, the potential for bias and the challenge of precision is well recognised [91]. It is particularly challenging for young children to estimate quantities of foods consumed and the role of parents is limited when children have consumed food separately from their parents. A smaller number of studies used direct observation and measurement by researchers, which are also limited in the capacity to assess all foods eaten throughout the day. The use of more objective measures to assess dietary intake, including the use of biomarkers and electronic shopping data can supplement and strengthen the conclusions that can be drawn from dietary self-report measures [92, 93]. These dietary self-report measures remain central to studies of nutritional interventions; hence attention to standardised reporting of these results facilitates the synthesis required in a systematic review. A further limitation was the inclusion of English language articles only, although only eight potentially eligible studies were excluded on the basis of language.

The evidence from this review indicates that schools have been shown to be an effective setting to improve primary school aged children’s dietary intake. Although the programs have been shown to be feasible to conduct on an ongoing basis, there is still limited evidence of the long-term benefits. Thus, careful evaluation of both the effectiveness and cost-effectiveness of such programs remains important. Given the moderate impact, it is relevant to consider how direct family-based strategies may be incorporated into these programs or operate concurrently in the same communities. Effective family-based programs have generally operated on a small scale and the feasibility of implementing these on a larger scale or multiple settings need further investigation.

## Conclusion

Family-based nutrition programs which support parents with simple nutrition information have demonstrated potential to substantially reduce dietary fat and increase F&V, while school-based nutrition programs have shown the potential to moderately increase F&V intake, particularly fruit. Effective components of the family-based programs have been simple dietary messages directed towards parents with regular follow-up, while the effective school-based programs have incorporated role-models including peers, teachers and heroic figures, rewards and increased access to healthy foods. However, there is limited evidence of the sustainability of effective family and school nutrition programs, particularly their impact on biomedical health outcomes. Given the limited impact of individual programs, complementary nutrition interventions are needed which build a supportive environment and provide the opportunities for everyone to eat healthily.

## Additional file

Additional file 1: Search Strategy-Medline version. (DOCX 17 kb)
