# Supplementary material for: How effective are family-based and institutional nutrition interventions in improving children’s diet and health? A systematic review
Source: BMC Public Health. 2017 Oct 17;17:818. doi: 10.1186/s12889-017-4795-5 (PMC5645887; doi:10.1186/s12889-017-4795-5)
Supplement: Additional file 1: — Search Strategy-Medline version. (DOCX 17 kb) [file 12889_2017_4795_MOESM1_ESM.docx]

Ovid Technologies, Inc. Email Service

------------------------------

Database: Ovid MEDLINE(R) In-Process & Other Non-Indexed Citations, Ovid MEDLINE(R) Daily and Ovid MEDLINE(R) <1946 to

Present>

Search Strategy:

--------------------------------------------------------------------------------

1 exp Food/ (1094645)

2 exp Health Food/ (1669)

3 exp Food Habits/ (151048)

4 exp Food Supply/ (8424)

5 Legislation, food/ (2079)

6 exp Nutrition Policy/ (6697)

7 food deprivation/ (8101)

8 food.mp. [mp=title, abstract, original title, name of substance word, subject heading word, keyword heading word,

protocol supplementary concept word, rare disease supplementary concept word, unique identifier] (421072)

9 Food choice.mp. [mp=title, abstract, original title, name of substance word, subject heading word, keyword heading

word, protocol supplementary concept word, rare disease supplementary concept word, unique identifier] (1098)

10 Food Preferences/ (10730)

11 Fruit/ (27907)

12 Vegetables/ (17483)

13 1 or 2 or 3 or 4 or 5 or 6 or 7 or 8 or 9 or 10 or 11 or 12 (1470372)

14 Financing, government/ (19258)

15 Family/ (63698)

16 family-based.mp. (5625)

17 Family Health/ (21288)

18 Schools/ (22289)

19 Schools, nursery/ (1291)

20 health education/ (53923)

21 child care/ (4803)

22 *child/ or *child, preschool/ (62367)

23 national health programs/ (26720)

24 food stamp*.mp. [mp=title, abstract, original title, name of substance word, subject heading word, keyword

heading word, protocol supplementary concept word, rare disease supplementary concept word, unique identifier] (312)

25 WIC.mp. [mp=title, abstract, original title, name of substance word, subject heading word, keyword heading word,

protocol supplementary concept word, rare disease supplementary concept word, unique identifier] (986)

26 supplemental nutrition assistance program.mp. [mp=title, abstract, original title, name of substance word,

subject heading word, keyword heading word, protocol supplementary concept word, rare disease supplementary concept

word, unique identifier] (99)

27 14 or 15 or 16 or 17 or 18 or 19 or 20 or 21 or 22 or 23 or 24 or 25 or 26 (271860)

28 13 and 27 (16208)

29 Nutrition Therapy/ (955)

30 Nutritional Status/ (24401)

31 nutrition.mp. (165512)

32 Diet/ec, sn [Economics, Statistics & Numerical Data] (3058)

33 "Outcome Assessment (Health Care)"/ (52517)

34 Birth Weight/ (33874)

35 nutritional physiological phenomena/ or exp child nutritional physiological phenomena/ or exp maternal

nutritional physiological phenomena/ or nutritional requirements/ (92854)

36 Health Status/ (63265)

37 health status disparities/ (8724)

38 "delivery of health care"/ (68602)

39 Healthcare Disparities/ (8623)

40 carotenoids/ or beta carotene/ (18569)

41 Biological Markers/ (172706)

42 plasma nutrient level*.mp. (17)

43 Folic Acid/ (21529)

44 Ascorbic Acid/ (36366)

45 Health Knowledge, Attitudes, Practice/ (77512)

46 Health Behavior/ (36606)

47 Risk Reduction Behavior/ (8418)

48 Healthy People Programs/ (982)

49 growth disorders/pc (488)

50 29 or 30 or 31 or 32 or 33 or 34 or 35 or 36 or 37 or 38 or 39 or 40 or 41 or 42 or 43 or 44 or 45 or 46 or 47 or

48 or 49 (792568)

51 28 and 50 (6723)

52 (Afghanistan or Bangladesh or Benin or Burkina Faso or Burundi or Cambodia or Central African Republic or Chad or

Comoros or Congo or Eritrea or Ethiopia or Gambia or Ghana or Guinea-Bissau or Haiti or Kenya or Kyrgyz Republic or Lao

or Liberia or Madagascar or Malawi or Mali or Mauritania or Mozambique or Myanmar or Nepal or Niger or Rwanda or Senegal

or Sierra Leone or Somalia or Tajikistan or Tanzania or Togo or Uganda or Uzbekistan or Vietnam or Yemen or Zambia or

Zimbabwe).ti. (69942)

53 (Albania or Angola or Armenia or Azerbaijan or Belize or Bhutan or Bolivia or Cameroon or Cape Verde or China or

Cote d'Ivoire or Djibouti or Ecuador or Egypt or El Salvador or Guatemala or Guyana or Honduras or India or Indonesia or

Iran or Iraq or Jordan or Kiribati or Kosovo or Lesotho or Maldives or Marshall Islands or Micronesia or Moldova or

Mongolia or Morocco or Nicaragua or Nigeria or Pakistan or Papua New Guinea or Paraguay or Philippines or Samoa or

Solomon Islands or Sri Lanka or Sudan or Swaziland or Syrian Arab Republic or Thailand or Timor-Leste or Tonga or

Tunisia or Turkmenistan or Ukraine or Vanuatu or West Bank or Gaza).ti. (142760)

54 (Algeria or American Samoa or Argentina or Belarus or Bosnia or Herzegovina or Botswana or Brazil or Bulgaria or

Chile or Colombia or Costa Rica or Cuba or Dominica or Dominican Republic or Fiji or Gabon or Grenada or Jamaica or

Kazakhstan or Latvia or Lebanon or Libya or Lithuania or Macedonia or Malaysia or Mauritius or Mayotte or Mexico or

Montenegro or Namibia or Palau or Panama or Peru or Poland or Romania or Russia or Serbia or Seychelles or South Africa

or St Lucia or Suriname or Turkey or Uruguay or Venezuela).ti. (114907)

55 Developing Countries/ (64458)

56 52 or 53 or 54 or 55 (375642)

57 (America$ or Andorra or Antigua or Barbuda or Aruba or Australia or Austria or Bahamas or Bahrain or Barbados or

Belgium or Bermuda or Brunei Darussalam or Canada or Cayman Islands or Channel Islands or Croatia or Cyprus or Czech

Republic or Denmark or Estonia or Equatorial Guinea or Faeroe Islands or Finland or France or French Polynesia or

Germany or Greece or Greenland or Guam or Hong Kong or Hungary or Iceland or Ireland or Isle of Man or Israel or Italy

or Japan or Korea or Kuwait or Liechtenstein or Luxembourg or Macao or Malta or Monaco or Netherlands or New Caledonia

or New Zealand or Northern Mariana Islands or Norway or Oman or Portugal or Puerto Rico or Qatar or San Marino or Saudi

Arabia or Singapore or Slovak$ or Slovenia or Spain or Sweden or Switzerland or Trinidad or Tobago or United Arab

Emirates or United Kingdom or United States or Virgin Islands).ti. (401094)

58 56 not 57 (367093)

59 51 not 58 (6048)

60 randomized controlled trial.pt. (400699)

61 controlled clinical trial.pt. (90738)

62 intervention studies/ (7544)

63 evaluation studies/ (206446)

64 program evaluation/ (48230)

65 random allocation/ or clinical trial/ or single-blind method/ or double-blind method/ or control groups/ (627236)

66 (randomized or randomised or placebo or randomly).ab. (646788)

67 trial.ti. (139130)

68 (time adj series).ab,ti. (17401)

69 quasi-experiment*.ab,ti. (6451)

70 (pre test or pretest or (posttest or post test)).ab,ti. (17187)

71 "before and after stud*".ab,ti. (1696)

72 controlled before.ab,ti. (754)

73 ((evaluat* or intervention or interventional) and (control or controlled or study or program* or comparison or

"before and after" or comparative)).ab,ti. (1586769)

74 ((intervention or interventional) adj5 evaluat*).ab,ti. (11073)

75 60 or 61 or 62 or 63 or 64 or 65 or 66 or 67 or 68 or 69 or 70 or 71 or 72 or 73 or 74 (2573041)

76 59 and 75 (1945)

77 limit 76 to yr="1980 -Current" (1918)

78 *Obesity/ (96024)

79 77 not 78 (1690)
